# Livestock Challenge Models of Rift Valley Fever for Agricultural Vaccine Testing

**DOI:** 10.3389/fvets.2020.00238

**Published:** 2020-05-27

**Authors:** Andrea Louise Kroeker, Shawn Babiuk, Bradley S. Pickering, Juergen A. Richt, William C. Wilson

**Affiliations:** ^1^Canadian Food Inspection Agency, National Centre for Foreign Animal Disease, Winnipeg, MB, Canada; ^2^Department of Immunology, University of Manitoba, Winnipeg, MB, Canada; ^3^Department of Medical Microbiology, University of Manitoba, Winnipeg, MB, Canada; ^4^Center of Excellence for Emerging and Zoonotic Animal Diseases (CEEZAD), Manhattan, KS, United States; ^5^USDA, Arthropod-Borne Animal Diseases Research Unit (ABADRU), Manhattan, KS, United States

**Keywords:** Rift Valley Fever virus, RVFV, vaccine, cattle, sheep, goat, deer, ruminants

## Abstract

Since the discovery of Rift Valley Fever virus (RVFV) in Kenya in 1930, the virus has become widespread throughout most of Africa and is characterized by sporadic outbreaks. A mosquito-borne pathogen, RVFV is poised to move beyond the African continent and the Middle East and emerge in Europe and Asia. There is a risk that RVFV could also appear in the Americas, similar to the West Nile virus. In light of this potential threat, multiple studies have been undertaken to establish international surveillance programs and diagnostic tools, develop models of transmission dynamics and risk factors for infection, and to develop a variety of vaccines as countermeasures. Furthermore, considerable efforts to establish reliable challenge models of Rift Valley fever virus have been made and platforms for testing potential vaccines and therapeutics in target species have been established. This review emphasizes the progress and insights from a North American perspective to establish challenge models in target livestock such as cattle, sheep, and goats in comparisons to other researchers' reports. A brief summary of the potential role of wildlife, such as buffalo and white-tailed deer as reservoir species will also be discussed.

## Background and Introduction

In the last 20 years there has been a re-emergence of various well-known arboviral diseases, many of which are zoonotic in nature, such as West Nile, Japanese encephalitis, Rift Valley fever, Venezuelan equine encephalitis, and Eastern equine encephalitis (1). Among these, Rift Valley fever (RVF) is considered a significant threat to animal and public health, economy, and food (2–4). Rift Valley fever was first reported in Kenya in 1930 (5), and has since created sporadic outbreaks in cattle and small ruminants with associated zoonotic spread to humans in sub-Saharan Africa. The disease first came to global attention during an outbreak in Egypt in 1977–78, involving at least 200,000 human infections (6). It was during the Egyptian outbreak when ocular degeneration was first associated with RVFV infections in humans (7). Global concerns were raised when RVF virus (RVFV) spread to Saudi Arabia and Yemen in 2000 (8) as well as during an outbreak in East Africa (Kenya, Tanzania, Somalia, and the Sudan) in 2006/7 (9–13). Since then there have been outbreaks in Madagascar (3), Mauritania 2010 (14), Namibia 2010 (15), South Africa 2008–2011 (16), Mozambique 2014 (17), Republic of Niger 2016 (18), and Kenya 2018 (WHO). The presence of the disease, especially during outbreaks, has significant socio-economic impact in endemic regions (19, 20). This along with the potential risk of RVFV importation into Europe and the U.S. (4) as well as its potential use as a biological weapon (21, 22), has led to intensified research on developing mitigation strategies. Developing such strategies requires a detailed understanding of the mosquito-transmitted virus that causes RVF disease.

RVFV is endemic in Sub-Saharan Africa and continues to cause sporadic outbreaks that are of veterinary and public health concern. Although RVFV is primarily a disease of domestic and wild animals, there can be spillover to humans during outbreaks than can lead in rare cases to lethal hemorrhagic disease in humans. RVFV outbreaks occur in 7–10 year cycles presumably as the number of seropositive animals in the population decrease, and/or ideal weather conditions for the mosquito vectors are present. It has also been suggested that recently emerged strains of RVFV might be more virulent to humans (23). Thus, RVF is clearly a disease that requires a “One Health” approach to mitigation strategies (24, 25) as it is a potential threat to animal and public health, economy, and food security (4, 25–27).

Outbreaks of RVF occur when conditions are ideal for mosquito expansion and virus transmission. *Aedes mcintoshi* mosquito species are thought to initiate epizootic outbreaks because of their transovarial transmission capability (28). Once the outbreak has been established, it can then be maintained by *Aedes* and other species (e.g., *Culex* and *Mansonia)* which can both replicate and transmit the virus (29). Although this is a well-accepted hypothesis for RVFV maintenance, transovarial transmission has only been demonstrated in one study. Alternatively, the mosquito to animal transmission cycle could be continuous at low levels and only become observed when ideal environmental conditions occur. The importance of understanding the potential role of transovarial transmission in mosquito-borne viruses has been reviewed (30). An increasing number of studies have also identified other species of mosquitoes that are either susceptible to RVFV and/or are capable of transmitting RVFV in the *Anopheles, Mansonia*, and other mosquito genera (13, 31). North American species such as *Aedes canadensis, Aedes taeniorhynchus*, and *Culex tarsalis* (32–34) and the stable fly species *Stomoxys calcitrans* (33) have also been shown to be capable of transmitting RVFV. The control of mosquitoes involved in RVFV transmission is complex because there are numerous mosquito species present in endemic and non-endemic areas that are capable of virus infection and transmission [reviewed in Linthicum et al. (29)], and continuous low-level transmission of RVFV to domestic and wild animals in endemic areas may also help maintain the virus. Other species that may play a role in RVFV ecology and have been reported to be susceptible to RVFV are mice, rats, shrews, dormice, and bats (35–40). Additional wild animal species that have been investigated include the African buffalo, primates, elephants, rhinoceros, deer, and coyotes (41–45). However, it is difficult to determine the role of susceptible wild animals in maintenance and transmission of RVFV. Based on a risk model, transmission and seroprevalence rates in both domestic and wild animals correlate positively with the risk of zoonotic infection of people (46).

RVFV is in the order *Bunyavirales* (*Phenuiviridae*; genus *Phlebovirus*), with a genome consisting of three negative-sense, single stranded RNA segments; L (large), M (medium), and S (small). The L segment encodes the RNA-dependent RNA polymerase (47). The M segment encodes the precursor protein of two structural glycoproteins, Gn and Gc, which are present on the virus surface. Cleavage of the precursor protein leads to two additional non-structural proteins of 78 kDa (P78 or LGp) and 14 kDa (NSm) in molecular mass (48–51). The Gn and Gc form heterodimers on the virus surface (52) and are involved in attachment of the virus to the host cell (53, 54). The NSm has been shown to inhibit apoptosis but is not essential for virus replication (55). Although also not critical for RVFV virulence, lack of NSm did reduce mortality and increase the number of animals demonstrating neurological disease in subcutaneously infected rats (56) and NSm mutated viruses were attenuated in intraperitoneally infected mice (57). The LGp/P78 protein, which is not associated with RVFV virulence in mice, is packaged into viruses grown in C6/36 (*Aedes albopictus)* insect cells, but not in mammalian cells, and is a major determinant of virus dissemination in mosquitoes (57, 58). Interestingly, additional studies showed that NSm is involved in virus replication and dissemination in *Aedes aegypti* mosquitoes (59, 60). The S segment utilizes an ambisense strategy encoding the nucleocapsid (N) protein in the anti-sense direction and the NSs protein in the sense direction (61). The N protein is the most abundant protein in the virion and plays a key role in transcription and replication and reconstitutes the ribonucleoprotein (RNP) complex together with the vRNA and the L protein (62). The N protein is immuno-dominant and is used as an antigen for diagnostic assays (63). The NSs protein has immunomodulatory functions and acts as interferon-antagonist via the inhibition of host gene transcription (64–66). The NSs protein is produced early during RVFV infection and has also a positive effect on viral replication and RNA transcription (67). The above described studies indicate that both, LGp/P78 and NSm seem important for virus maintenance in mammalian and insect hosts, and that NSs is an important virulence factor. This information led to the development of a NSm and NSs double deleted virus that was shown to be attenuated in rats (68). When used as a vaccine, RVF virus containing NSm and NSs deletions were shown to be safe and non-teratogenic in pregnant sheep as well as protective against the development of viremia and RVF disease (69). These findings are supportive of the mechanistic studies in but since just rodent model systems; however, there have been only a few studies directed at understanding of the molecular basis of RVFV virulence and molecular pathogenesis in target livestock species.

RVFV research conducted in target livestock species has been primarily focused on the development of diagnostics and vaccines. As a result of the 2006/2007 RVF outbreak in Kenya (12, 70), this disease garnered increased attention from global scientific communities. This has led to an increased focus on identifying North American mosquito species that are capable of being infected and able to transmit RVFV, as well as improving RVFV diagnostic tools (71–75), developing better risk models for RVF (76–79) and evaluating these models using seroprevalence data (46). Several studies have also published data demonstrating that RVFV circulates in endemic countries during inter-epidemic periods (43, 80, 81).

Currently, there are modified live virus (MLV) or attenuated (82, 83) and inactivated/killed vaccines licensed for veterinary use in RVFV endemic countries and one attenuated MLV vaccine with a conditional license in the United States (USDA-APHIS, CVB Notice 13-12). Since various RVFV vaccine approaches were recently reviewed in several publications (25, 84–86), they will be only discussed briefly here. Other recent reviews have focused on the molecular biology, reassortment capacity, diagnostics, and vaccines (86, 87). Thus, this review will focus on the development of target livestock infection models.

## Developing RVFV Infections in Target Species

The animals most susceptible to RVFV consist of ruminants such as sheep, goats and cattle, as well as camels, buffaloes, and humans (88). These animals all produce viremia upon infection with clinical signs that typically range from asymptomatic to moderate and high severity to death; in addition, pregnant animals suffer from high rates of abortion (17, 89–91). Although the documentation of confirmed RVFV cases, deaths and abortions in animals has been sparse, recent studies with confirmed RVFV antibody status have provided estimates of animal mortality rates during different outbreaks [(92); see Table 1].

**Table 1:** Rates of mortality in animals during recent RVFV outbreaks.

	**Cattle**	**Sheep**	**Goats**	**Camels**	**Buffaloes**	**Wild species**
Niger 2016 (18)	X					
Mozambique 2014 (17)	x	8 abortions; 7 neonatal deaths	42 abortions; 5 neonatal deaths	x	x	X
Senegal 2013 (93)	0% mortality	x	19–33% mortality	x	x	37.5% mortality
South Africa 2010–11^*^ (94)	61% mortality	62% mortality	55% mortality	100% mortality	100% mortality	100% mortality
Kenya 2006–07 (12)	14% mortality			X	x	X
Mauritania 2003 (9)	x	70% abortions		X	x	X
Mauritania 1998 (95)	4.6% abortions	9.7% abortions	47.4% abortions	20.6% abortions	x	X

* *The field-study sites were selected for farms that had high numbers of deaths (96)*.

*%mortality, deaths/confirmed cases; x, no data available*.

Early work on RVFV livestock infections was done in South Africa (89, 90, 97), and a few RVFV experimental infections of livestock were conducted in the 1970/80s at the Plum Island Animal Disease Center (USDA) (98). These early studies included safety and efficacy trials for both inactivated (98) and a mutagen-attenuated MLV vaccine (99, 100). No RVFV infection studies using livestock had been conducted on the mainland of North America since these earlier studies. In 2006, the Canadian Food Inspection Agency (CFIA) and the United States Department of Agriculture (USDA) were tasked with developing target animal infection models to develop and evaluate diagnostic and control strategies. The procedures and models developed through this cooperative research were then transferred to the Biosecurity Research Institute (BRI) at Kansas State University (KSU) through collaboration with the Center of Excellence for Emerging and Zoonotic Animal Diseases (CEEZAD) at KSU. The results of this ongoing three-way collaboration are reviewed here and discussed within context of the literature.

There are several traits desirable in a veterinary RVFV vaccine, but at a minimum the vaccine should protect against abortions in pregnant animals and should prevent viremia to avoid transmission. In addition, the vaccine should be safe and efficacious in the most vulnerable target species and groups, that is, in fetal and newborn animals. For example, RVFV can be transmitted vertically from pregnant ewes to their fetus (101). Newborn lambs also remain highly susceptible to RVFV after they have been weaned as they lose any protection from maternally-derived antibodies. As weaning can occur any time between 3 weeks and 4 months, the earlier a vaccine can be given, the better. Therefore, in developing challenge models, these are important aspects of the disease that should be considered. For example, previous studies have utilized pregnant ewe models to evaluate whether experimental attenuated or MLV vaccines cross the placenta (102) and are safe to administer during pregnancy. Although this model is highly susceptible to RVFV and is useful for evaluating vaccine safety, using pregnant animals in high containment animal rooms (i.e., BSL-3Ag) is logistically challenging. Therefore, alternative models have also been developed and will be discussed.

## Sheep Model Development

Several challenge models have been developed in 2–3 month old lambs that display significant pathology that is typical of RVFV. For example, Kortekaas et al. has utilized intravenous (103–105) and intraperitoneal inoculation (106) of the recombinant 35/74 RVFV isolate grown in mammalian cells in Texel, Romane, and other European breeds at 10^5^ TCID_50_. The challenge controls in these studies developed peracute clinical signs and fever, viremia for 4–6 days with peak titers of 10^5^-10^6^ TCID_50_, and virus was detected in the liver. Clinical markers also indicated elevated levels of plasma alkaline phosphatase and alanine transferase (hepatic dysfunction) as well as blood urea nitrogen and creatinine (renal dysfunction) (106). Pathology varied between different individual animals as is typically seen in studies with ruminants, but heavily affected the liver, and could also include abdominal hemorrhage, pulmonary edema and petechiae in the spleen, heart and lungs (104). Notably, these studies indicated an overall rate of 20% mortality with up to 70% mortality in some studies. Other pathological findings have been found in a study where 2–3 month-old lambs were infected with various field isolates (56/74, 252/75, AN1830, AR20368) and included the development of corneal opacity (107). A study with the Zinga isolate also induced severe clinical signs characterized by hyperactivity, watery and mucoid nasal discharges, projectiles and bloody diarrhea, external hemorrhage and neurological signs (108).

Other groups have developed 2–3 month old challenge models with the 56/74 RVFV isolate in Ripolessa (107, 109), Colmenarena (110) and other (111) sheep breeds using subcutaneous inoculation at 10^5^-10^6^ TCID_50_. Similarly, these animals also developed clinical signs and fever and viremia for 4–5 days with peak titers of 10^5^-10^6^ TCID_50_. Interestingly, two of these studies detected oral and nasal shedding of viral RNA between one and 7 days post infection (107, 109) with a few samples also leading to viral isolation (109); one of the uninfected sentinel animals even became seropositive suggesting that horizontal transmission may have occurred. The only other study to report horizontal transmission in sheep is after challenge with the Zagazig strain (98). While the studies by Busquets utilized passage 12 virus stocks grown in mammalian cells and had no deaths, the two studies by Chrun and Lorenzo had ~20% mortality and both used passage 5 mosquito-cell derived viral stocks. Since these three studies were performed independently in different breeds of sheep, it is not possible to directly compare these results; however, it is interesting to note that the age, viral strain, route, and dose were all similar in these studies, leaving breed, source of virus, and virus passage history as the main differences.

Challenge models have also been developed in older lambs at 4–6 months of age. For example, Suffolk and Arcott-Rideau breeds were challenged subcutaneously with 10^5^ or 10^7^ pfu of the ZH-501 RVFV isolate and compared virus stocks that had been grown in either mammalian Vero or mosquito C6/36 cells (112). While the mosquito-cell grown virus produced a robust and consistent infection, the mammalian-cell produced virus had reduced efficacy with viremia only present on day 1 and lower titers (10^2^ pfu/ml serum) (112). In contrast to the acute illness seen in the 2–3 month old lambs, 4–6 month old sheep only produced a mild, self-resolving disease with transient pyrexia during the first week after infection and had no obvious gross pathology at 7 days post infection.

At KSU, 4–6 month-old Dorper-Katahdin and Polypay sheep were inoculated subcutaneously with 10^6^ pfu passage-2 mosquito-derived virus stock using either RVFV isolate Kenya-128B-15 (Ken06) or SA01-1322 (SA01) (113). SA01 originated from the Saudi Arabian outbreak in 2001 (8) and Ken06 from the Kenyan outbreak in 2006 (114) which had affected an unusually large number of people and was speculated to possibly be more pathogenic. Both RVFV strains produced detectable viremia between days 1 and 5, and both strains produced gross pathology and histopathology consistent with RVFV virus infection at between days 3–5 (113). However, the Ken06 group tended to have higher viremia and serum aspartate aminotransferase (AST) levels indicating that liver damage was significantly higher in Ken06 infected lambs as compared to SA1 infected animals. Virus isolation from nasal swabs detected infectious virus in a three out of six animals infected with the Ken06 strain while no shedding was seen in SA01-infected lambs. Histopathology and viral antigen was detectable in a wide variety of organs including the spleen, liver, adrenal gland, and kidney during the first week after infection, although no specific differences were attributed to one isolate compared to the other. In addition, histopathology without antigen staining was detected in the brain, intestine, and the eye at later time points, and infectious virus could also be isolated from several tissues including the spleen and the liver between 3–5 days post challenge (25, 113). Three of five animals inoculated with Ken06 had large necrotic foci in the liver, hemorrhage in the liver and spleen, and pulmonary edema.

A summary of the discussed sheep models can be found in Table 2.

**Table 2:** Summary of included sheep studies involving RVFV challenge.

**Breed**	**Age**	**Virus isolate**	**Route**	**Infection dose**	Cells^*^	**Viremia length**	**Viremia peak**	Organs infected^**^	Clinical chemistry^***^	**Shedding**	**Deaths**	**References**
Dorper-Katahdin X	4–5m	SA01	SC	10^6^ pfu	I	2d	10^4^ pfu/ml	B, L, S, H	BUN	No	N	(113)
Dorper-Katahdin X	4–5m	Ken06	SC	10^6^ pfu	I	5d	10^7^ pfu/ml	B, L, S, H	AST, BUN	Nasal VI	No	
Polypay	4–5m	Ken06	SC	2 × 10^6^ pfu	I	4d	10^7^ pfu/ml	L, S	AST, BUN	n.a.	3/5	
Rideau-Arcott	4–6m	ZH501	SC	10^5^ pfu	M	3d	10^5^ pfu/ml	n.a.	n.a.	n.a.	No	(112)
Rideau-Arcott	4–6m	ZH501	SC	10^5^ pfu	I	2d	10^5^ pfu/ml	n.a.	n.a.	n.a.	No	
Rideau-Arcott	4–6m	ZH501	SC	10^7^ pfu	M	1d	10^3^ pfu/ml	n.a.	n.a.	n.a.	No	
Rideau-Arcott	4–6m	ZH501	SC	10^7^ pfu	I	4d	10^5^ pfu/ml	n.a.	n.a.	n.a.	No	
Dorper	4–6m	56/74	SC	10^6^ pfu	n.a.	n.a.	n.a.	n.a.	n.a.	n.a.	No	(115)
Texel-X	2–3m	rec35/74	IV	10^5^ TCID_50_	M	n.a.	n.a.	L,S	n.a.	n.a.	3/7	(105)
Romane	2–3m	rec35/74	IV	10^5^ TCID_50_	M	n.a.	10^6^ TCID_50_	L, S, K, A, Lu, LN	n.a.	n.a.	3/8	(103)
Texel	2–3m	rec35/74	IV	10^5^ TCID_50_	M	n.a.	n.a.	L, B	n.a.	n.a.	1/8	(104)
European	6 wks	rec35/74	IP	10^5^ TCID_50_	M	9d	10^5^ TCID_50_	L	ALP, CK, BUN	n.a.	1/6	(106)
n.a.	2m	56/74	SC	10^6^ TCID_50_	I	5d	10^6^ TCID_50_	n.a.	n.a.	n.a.	2/8	(111)
Colmenarena	3m	56/74	SC	10^5^ TCID_50_	I	4d	10^6^ TCID_50_	n.a.	BUN, ALB	n.a.	1/5	(110)
Ripollesa	2–3m	56/74	SC	10^5^ TCID_50_	M	4d	10^6^ TCID_50_	n.d.	n.a.	Nasal and oral VI	No	(109)
Ripollesa	2–3m	56/74	SC	10^5^ TCID_50_	M	n.a.	n.a.	K	n.a.	Nasal and oral RNA	No	(107)
Ripollesa	2–3m	252/75	SC	10^5^ TCID_50_	M	n.a.	n.a.	K	n.a.	Nasal and oral RNA	No	
Ripollesa	2–3m	AN1830	SC	10^5^ TCID_50_	M	n.a.	n.a.	K	n.a.	Nasal and oral RNA	No	
Ripollesa	2–3m	AR20368	SC	10^5^ TCID_50_	M	n.a.	n.a.	K	n.a.	Nasal and oral RNA	No	

* *M, mammalian cell culture; I, insect cells culture*.

** *B, brain; L, liver; S, spleen; H, heart; Lu, lung; LN, lymph node; K, kidney; A, adrenal gland*.

*** *ALP, alkaline phosphatase; CK, creatinine kinase; BUN, blood urea nitrogen*.

## Goat Model Development

Initial experimental goat infection studies were performed with RVFV strain ZH501 (112) and were intended to establish the dose required for the induction of viremia, the timing of viremia and a comparison of inoculation virus grown in mammalian and insect cells. In this study, viremia occurred very quickly in goats, appearing on the first day post infection after SQ inoculation and lasted 2–5 days. By comparing an inoculation dose of 10^5^ pfu and 10^7^ pfu of the ZH-501 strain (112) we determined that the higher dose achieved more robust and reliable titers. A second study in Boer goats focused on the characterization of innate and adaptive immune responses in the blood after RVFV infection (116). Flow cytometry indicated that after RVFV infection there was a decline in CD5+ (T cells), CD172+ cells (monocytes and dendritic cells), and CD8+ T cells (cytotoxic T lymphocytes) and an increase in CD21+ cells (B cells). Interestingly, these effects were more pronounced in goats infected with mosquito cell-grown virus compared to goats infected with mammalian cell-grown virus (116). In addition, cytokine profiling in the blood demonstrated an increase of interleukin-12 at 1 dpi, an increase of IFN-γ at 2 dpi, and a steady increase of TNF-α, IL-6 and IL-1β up until the end of the observation period at 21 dpi (116). A Kenyan RVFV isolate (“Ken-UAP,” Genbank #MH175203.1, MH175204.1, MH175205.1) that had been proposed to be more pathogenic than ZH501 has also been tested in goats (117). Although the Ken-UAP and ZH501 RVFV isolates were not compared directly in the same goat breed, viremia titers after subcutaneous inoculations of each were comparable (112, 117).

Several novel alternative routes of infection have also been explored. For example, numerous manuscripts describing arbovirus infections have demonstrated that mosquito saliva can modulate the pathogenicity of the virus upon infection (118–123). To test the effect of saliva on RVFV infection in goats, we mimicked a methodology developed by Le Coupanec et al. (119) in mice; first, we allowed naïve mosquitoes to feed at a shaved site on the goats' skin and second, we then injected a known amount of virus subcutaneously (SQ) into the same area. Although the “mosquito-SQ” infection did not result in significant differences in viremia or antibody titers when compared to the “only SQ” infection, we noted that the “mosquito SQ” group retained higher levels of viral RNA in tissues at 28 days post challenge (117). Interestingly, viremia is delayed by 1 day when the virus is inoculated intranasally, suggesting that it has a longer transition (i.e., 48 h) route to travel to the bloodstream (117). Seroconversion kinetics are similar to that in sheep and cattle, occurring at 4–5 days post infection, and producing robust antibody titers at 21–28 days post infection. The tissues that are infected by RVFV may differ with breed, route of inoculation, and RVFV strain. However, spleen, liver, and lymph nodes are consistently positive and reliable targets for RVF diagnosis (112, 117). Other tissues that may be infected by RVFV include a variety of CNS regions such as the olfactory bulb, the trigeminal nerve, the cerebellum, the midbrain and the brainstem. The development of clinical signs varied from asymptomatic to mild and most commonly consisted of fever and diarrhea. To assist in identifying and quantifying other more subtle signs of disease, a RVFV clinical scoring sheet was developed for ruminants (see Supplementary Table 1). Using the clinical scoring sheet, subcutaneous infection of Nubian goats demonstrated slightly higher clinical scores than intranasal infection (Supplementary Figures 2A,C). In contrast, LaMancha goats had a higher clinical score after intranasal infection when compared to a subcutaneous infection, and a higher clinical score when infected with mosquito cell grown virus compared to mammalian cell grown virus (Supplementary Figures 2E,F). Different clinical outcomes after RVFV infection are also seen amongst different experimental groups with Boer goats remaining almost asymptomatic with mild clinical signs with ZH501, whereas Nubian and LaMancha goats exhibited clear clinical signs with Ken06 (Supplementary Figures 2A–I).

A few other goat breeds have been successfully used for RVFV model development or RVFV vaccine testing including the Galla (115) and Saanen (124) goat breed. Both experiments produced viremia but no clinical signs.

A summary of the discussed goat models can be found in Table 3.

**Table 3:** Summary of included goat studies involving RVFV challenge.

**Breed**	**Age**	**Virus isolate**	**Route**	**Infection dose**	Cells^*^	**Viremia length**	**Viremia peak**	Organs infected^**^	**Clinical chemistry**	**Shedding**	**Deaths**	**References**
BoerX		ZH501	SC	10^7^ pfu	M	n.a.	n.a.	n.a.	n.a.	n.a.	No	(116)
BoerX		ZH501	SC	10^7^ pfu	M	2d	10^3^ pfu/ml	n.a.	n.a.	n.a.	No	(112)
Galla	4–6m	56/74	SC	10^7^ pfu	I	n.a.	10^4^ pfu/ml	n.a.	n.a.	n.a.	No	(115)
Nubian	4–6m	KenUAP	SC	10^7^ pfu	I	3d	10^3^ pfu/ml	M, P, R, Olf, Tri, CB, MB	n.a.	Nasal RNA	No	(117)
Nubian	4–6m	KenUAP	mosSC	10^7^ pfu	I	3d	10^3^ pfu/ml	M, P, R, S, L, Olf, Tri, BS, CB, MB, Cer	n.a.	Nasal RNA	No	
Nubian	4–6m	KenUAP	IN	10^7^ pfu	I	3d	10^5^ pfu/ml	M, P, R, S, Olf, Tri, MB, CB, MB	n.a.	Nasal RNA	No	
LaMancha	4–6m	KenUAP	SC	10^7^ pfu	I	2d	10^3^ pfu/ml	No	n.a.	No	No	
LaMancha	4–6m	KenUAP	IN	10^7^ pfu	I	2d	10^3^ pfu/ml	No	n.a.	No	No	
LaMancha	4–6m	KenUAP	IN	10^7^ pfu	M	2d	10^3^ pfu/ml	No	n.a.	No	No	

* *M, mammalian cell culture; I, insect cells culture*.

** *L, liver; S, spleen; H, heart; Lu, lung; M/P/R, mesenteric/prescapular/retropharyngeal lymph node; Olf, olfactory bulb; Tri, trigeminal ganglion; CB, cerebellum; Cer, cerebrum; MB, midbrain; BS, brainstem*.

## Cattle Model Development

At KSU, an initial cattle model was established using an Angus or Hereford cross (125), which are commonly bred in North America or Europe for beef production and could be sourced from local farms. Similar in design to our study with sheep, a subcutaneous injection of mosquito-cell grown Kenya-128B-15 (Ken06) or SA01-1322 (SA01) at a titer of 10^6^ pfu was inoculated (125). There was variation in the responses to RVFV infection. Most of the infected animals had detectable viremia at least 1 day post infection (4 of 5), but some were asymptomatic, some were febrile and one animal died of infection. There was detectable virus in nasal swabs during the peak of viremia but no evidence of contact transmission to the contact control animals (125).

In an effort to increase the reliability of the infection in cattle, a second study was undertaken in Holstein calves in which we used three different routes of infections, including intradermal, intranasal and a combination of subcutaneous, intradermal, and intranasal. Despite an adherence to subcutaneous RVFV infections in a majority of manuscripts, we tested whether an intradermal challenge model could result in an enhanced clinical course of RVFV infection. Our results indicated that the degree of viremia was similar to that of a subcutaneous infection, although far fewer tissues tested positive for virus presence in the intradermal model. After intradermal inoculation, infectious virus was only found in turbinates, prescapular lymph nodes, and retropharyngeal lymph nodes (126). Although ruminants are not known to become infected intranasally in the wild, they are quite susceptible to intranasal infection. Intranasal inoculation of cattle led to high titers of viremia with peak titers of 6 × 10^5^ pfu/ml blood and produced infectious virus in a variety of tissues including spleen, liver, kidney, lymph nodes, heart, thyroid, turbinates, and cerebellum (126). We speculated that during intranasal and subcutaneous/intradermal RVFV inoculations, the virus may follow different pathways to reach the bloodstream (117). This led us to hypothesize that combining these three routes could produce an additive effect and increase viremia. However, when cattle were infected using the intranasal, intradermal, and subcutaneous inoculation routes at the same time, this method produced less viremia than using each route individually. All three routes of infection all produced viremia in all animals as well as mild but observable clinical signs such as fever, a depressed disposition, and a lessened appetite in some animals (Supplementary Figure 1). Viral RNA was detected in nasal swabs but no infectious virus was present (126).

In a third study, Warimwe et al. challenged 4–6 month-old Holstein-Friesian cattle with 10^7^ pfu RVFV 56/74IN subcutaneously. Similar to the experiments performed at NCFAD and KSU, control cattle developed fever and viremia, with peak viremia levels of 10^5^ relative pfu (115).

A summary of the discussed cattle models can be found in Table 4.

**Table 4:** Summary of included cattle studies involving RVFV challenge.

**Breed**	**Age**	**Virus isolate**	**Route**	**Infection dose**	Cells^*^	**Viremia length**	**Viremia peak**	Organs infected^**^	Clinical chemistry^***^	**Shedding**	**Deaths**	**References**
Holstein	4–6m	56/74	SC	10^7^ pfu	I	n.a.	10^5^ pfu/ml	n.a.	n.a.	n.a.	No	(115)
Hereford-Angus	4–5m	SA01	SC	2 × 10^6^ pfu	I	2d	10^3^ pfu/ml	B, K, S, L	ALP		No	(125)
Hereford-Angus	4–5m	Ken06	SC	2 × 10^6^ pfu	I	4d	10^3^ pfu/ml	B, K, S, L	ALP		No	
Holstein	3–6m	KenUAP	ID	10^7^ pfu	I	2d	10^3^ pfu/ml	S, P, R, Tu	ALP, ALB	No	No	(126)
Holstein	3–6m	KenUAP	IN	10^7^ pfu	I	4d	10^6^ pfu/ml	M, R, S, L, K, Lu, Tu, Tr, I, H, BS, MB, CB, CSF	ALP, ALB, BUN	No	No	
Holstein	3–6m	KenUAP	SC-ID-IN	v	I	1d	10^2^ pfu/ml	L, Tu, Olf, Tri	ALP, ALB	Nasal RNA	No	

* *M, mammalian cell culture; I, insect cells culture*.

** *B, brain; L, liver; S, spleen; H, heart; Lu, lung; R/P/M, retropharyngeal/prescapular/mesenteric lymph node; K, kidney; Tu, turbinate; Tr, trachea; BS, brainstem; MB, midbrain; CB, cerebellum; CSF, cerebral spinal fluid; Olf, olfactory bulb; Tri, trigeminal ganglion; I, ileum*.

*** *ALP, alkaline phosphatase; ALB, albumin; BUN, blood urea nitrogen*.

## Deer

The role of wildlife as maintenance hosts has been and continues to be a concern in endemic regions in Africa and the Arabian Peninsula (43, 46), and is also of critical significance if the RVFV emerges in other, previously non-endemic regions. To predict the potential of North American wildlife to act as RVFV maintenance hosts, a panel of available wildlife-based cell lines were assessed for RVFV susceptibility (42). Cells derived from white-tailed deer (WTD, *Odocoileus virginianus*), an important wildlife species in North America, were found to be susceptible to RVFV infection. The abundance and wide distribution of WTD in North America and their known susceptibility to other vector-borne diseases is a serious concern (127–129). Risk models for RVFV have also addressed the issue of the potential role that WTD could play if RVFV were introduced to North America (130–133). To address this concern, a group of young, farm-reared WTD were experimentally infected with 10^6^ pfu of the Ken06 RVFV strain at the KSU BSL-3Ag facility using specially designed and constructed pens. Surprisingly, WTD were found to be highly susceptible to RVFV infection with lethality in two of the four animals after subcutaneous inoculation. A sentinel contact control animal, which was co-housed with the principally infected deer also became RVFV infected and had to be humanely euthanized due to severe clinical signs. All dead/euthanized animals had severe clinical signs including bloody diarrhea, which most likely caused the transmission of the virus via the oronasal route to the uninfected contact control animal (134).

## Overview on Factors Influencing RVFV Infection in Ruminants

- Age: The age of the animal is arguably one of the most important parameters to consider in a RVFV challenge model. All young ruminants (<3 months) are highly susceptible to RVFV infection and typically succumb to acute liver failure (89, 135–137). Recent studies at KSU and NCFAD have opted to develop challenge models in animals old enough to be weaned (4–6 months) due to the logistics of working in high containment (112, 113, 117, 125, 126). However, numerous vaccine studies have also successfully used younger animals at 2–3 months of age (104) as well as pregnant ewes to induce protective immunity (138).
- Isolate and passage history: Experiments at CFIA used a low passage ZH-501 strain kindly provided by Stuart Nichol, Centers for Disease Control, Atlanta, GA as well as a human isolate from the Kenya 2006/7 outbreak provided by Health Canada. At KSU, a mosquito isolate (SA01-1322) from the Saudi Arabia outbreak in 2001 was provided by Barry Miller CDC Fort Collins, CO through Richard Bowen Colorado state university and the Kenya 2006 strain was also a mosquito isolate (13); both isolates were propagated twice in Vero cells and twice in on mosquito C6/36 cells (8).
  South African researchers have used two other strains of RVFV in a vaccine efficacy trial (82). The first RVFV strain was Buffalo/99/MB/CER, isolated from an aborted fetus from an outbreak in 1998, and the second strain was the reference strain RVF 35-74, isolated from a sick sheep from an outbreak in 1974. Both virus strains were isolated using intra-cranial inoculation of mice plus one passage in BHK cells. Subcutaneous infection of sheep with 10^6^ pfu of the Zimbabwean strain of RVF produced viremia by 4 days post-infection (dpi) and caused transient fever, viraemia, leucopaenia, relative thrombocytopaenia, haemoconcentration and raised serum enzyme levels that indicated the development of necrotic hepatitis and virulence (139).
  In another study evaluating an adenovirus-based vaccine in Kenya, researchers used the RVF 56/74IN strain propagated in C6/36 cells and purified before challenge. This virus strain caused clinical disease and viremia following subcutaneous inoculation of a rather high dose of 10^7^ pfu per animal (115). In addition, a variety of field isolates have been compared in sheep including RVFV strains 56/74, 252/75, AN-1830 and AR-20368 (107), 1678/78 and Lunyo (139) and Zinga (108).
  Overall, there are very few studies that directly compare different RVFV isolates to each other and the question has been raised of whether RVFV virulence is increasing (23). Although the genetic variation among strains of RVFV is very small (≤ %5), there are still clear genetic lineages and distinct clades from outbreaks (140– 143). Because RVFV appears to circulate between vectors and naïve animals during the inter-epidemic periods (81), which could affect virus genetic population variation, it would also be beneficial to understand what effect this has on the virus' virulence. Faburay's study demonstrated distinct virulence between two outbreak isolates in livestock that suggest that Ken06 has increased virulence over SA01. Egyptian RVFV strains were shown to be almost ten-fold more virulent than sub-Saharan African strains in rats (144). Differences ranging between 50 and 90% mortality were also demonstrated in a mouse model (143). Studies are needed to confirm that these phenotypic differences are also observed in target livestock species and if genetic characteristics could be correlated.
- Cell line: Perhaps one of the most intriguing aspects of arbovirus infections is that the virus's pathogenicity can be changed depending on whether the inoculum is produced in a mammalian or insect host cell. This phenomenon was first characterized *in vitro* for alphaviruses (145) and then also for RVFV (146); importantly, we were able to demonstrate that these source effects also apply to RVFV infections *in vivo* (112). In addition, we could show that RVFV which is grown in mosquito cells incorporates the viral P78 protein into its virions, i.e., P78 is a structural protein of RFV viruses produced in insect cells. In contrast, P78 is not found in the virion when the virus is grown in mammalian cells (58). We proposed that the p78 present in virions of mosquito-grown viruses may function as a type I interferon antagonist, which may allow for a productive infection of initial target cells (58), as was shown earlier for alphaviruses (145).
- Route of inoculation: In addition to the experimental infections performed at NCFAD and KSU, other studies have shown that viremia can be induced by a wide variety of different routes including intramuscular (IM), intravenous (IV), intraperitoneal (IP), intracerebral (IC), subcutaneous (SQ), conjunctival, and oral inoculations (112, 137, 147–149). Subcutaneous injection consistently produced clinical responses and is easy to administer under BSL-3Ag conditions thus is a common method of administration (113, 125).

## Future Directions and Unanswered questions

In the field, the infecting dose of RVFV is widely variable ranging from a single mosquito bite which may contain a low level of infectious virions to animals being fed on repeatedly by numerous RVFV-infected mosquitoes, potentially resulting in inoculation with high levels of infectious virions. Understanding the minimal infectious dose required for infection in ruminants and humans is currently an area requiring further investigation. In addition, it is not well-understood what the effect of mosquito saliva has on *in vivo* RVFV infection or pathogenesis, especially in ruminants. In containment and field studies, inoculations such as subcutaneous injections are used to mimic a mosquito bite; however, the differences between a natural infection and a subcutaneous (or intradermal) injection are not well-characterized.

Moreover, it has recently been shown that goats (117) and cattle (126) produce robust viremia when experimentally inoculated through the intranasal route. This is not necessarily unexpected since humans can be infected by inhaling aerosols produced during animal slaughtering of infected livestock (91), but the differences between an intranasal and subcutaneous infection are not well-understood. While intranasal RVFV infection can produce severe encephalitis in rodents (102), NHPs (150) and people, it did not create any neurological disease in goats (117). However, the neurological effects of intranasal infection in cattle are still unknown, as the study was terminated at peak infection at 4 dpi. Therefore, it would be interesting to also characterize the intranasal infection in cattle over a longer period of time.

The transmission of RVFV in livestock in the absence of mosquitoes is also not fully understood and there have been conflicting reports. Although it was demonstrated in sheep that transmission could occur through contact exposure (98, 109), more recent studies have not demonstrated transmission from subcutaneously infected lambs to naïve or immunosuppressed lambs (105). One possible explanation for these conflicting results is that transmission between animals requires a minimum virus dose which likely varies between different virus strains. Alternatively, upper respiratory infections with parainfluenza 3 virus, adenoviruses, reoviruses, infectious bovine rhinotreacheitis virus, maedi-visna virus, sheeppox virus, goatpox virus, peste des petits ruminants virus or *Mycoplasma spp*. could cause perturbations in the nasal mucosa which could possibly enhance transmission efficiency in the field but are usually absent in laboratory experiments. The potential role of urine in transmission or RVFV has recently been highlighted by a study that isolated RVFV from the urine of an infected person (151), and it is possible that milk may serve as a source of RVFV transmission (152) to animal offspring and human consumers; both of these areas require further investigation. Mosquito transmission has been considered the primary route of exposure for livestock and wildlife, but not for humans. Additional investigations of alternative routes of exposure will provide further insights into the infectability of different RVFV strains and might allow correlation of phenotype with genotype.

The selection of mosquito cell line propagated virus for livestock inoculation studies at KSU was based on the observation of more consistent viremia in our early studies. The importance of the p78 protein in the insect vs mammalian hosts has been reported (57, 58, 60) but mechanistic understanding of how this might affect virulence in the vertebrate host has not been determined yet. Studies are also needed to examine whether the individual host animal or cell selects for specific genotypes, and how that might affect viral maintenance and virulence in both the vertebrate and invertebrate host. For example, the basis of the increased virulence of RVFV in recent outbreaks is yet unknown. So far, Sanger sequencing of many RVFV isolates has demonstrated a surprisingly high stability of RVFV (140, 142) however, there are no studies on the quasi-species variation of RVFV within a specific virus population either over a period of several days in a target animal or during and after several passages in the same or different animals. Such information would be important to understand the relative fitness and overall replication ability of RVF viruses.

It was recently determined that there is low level transmission during the inter-epidemic period (81); whether this is the only mechanism of viral maintenance is not clear yet. Also, how low level transmission restricts viral evolution is not known. Similarly, there is empirical evidence that various animal breeds have different susceptibilities to RVFV infection. However, well-designed controlled studies are needed to substantiate this observation. If confirmed, this could change husbandry techniques in RVFV endemic areas, and not only improve animal health but also have a significant effect on public health.

## Conclusion

The renewed interest in RVFV since the 2006/7 outbreak in Kenya has resulted in many advances in our basic knowledge about RVFV replication strategies and molecular pathogenesis in small animal and livestock models. It also has resulted in novel vaccine candidates and novel experimental challenge models as discussed in this review. In addition, novel tools for the detection of viral nucleic acids and antibodies, both for laboratory and point-of need use have been developed. These recent advances in RVFV mitigation strategies will allow a more rapid and effective control of the disease; unfortunately, the availability of these tools in endemic areas is rather limited (84). There are still many questions about the mechanisms and factors affecting viral maintenance and virulence. The models described here provide a good basis for developing studies to investigate these factors. There are still many questions to be addressed in RVF biology and epidemiology as discussed above in this review. We hope that the RVFV livestock models described here in detail will provide a sound basis for the design of studies to investigate these yet unknown questions.

## Author Contributions

WW, JR, and AK conceived the review topic. AK and WW prepared and wrote the manuscript. JR, SB, and BP critically reviewed the manuscript.

## Conflict of Interest

The authors declare that the research was conducted in the absence of any commercial or financial relationships that could be construed as a potential conflict of interest.
