# Supplementary material for: Livestock Challenge Models of Rift Valley Fever for Agricultural Vaccine Testing
Source: Front Vet Sci. 2020 May 27;7:238. doi: 10.3389/fvets.2020.00238 (PMC7266933; doi:10.3389/fvets.2020.00238)
Supplement: Supplementary file 1 [file Presentation_1.PPTX]

## Slide 1
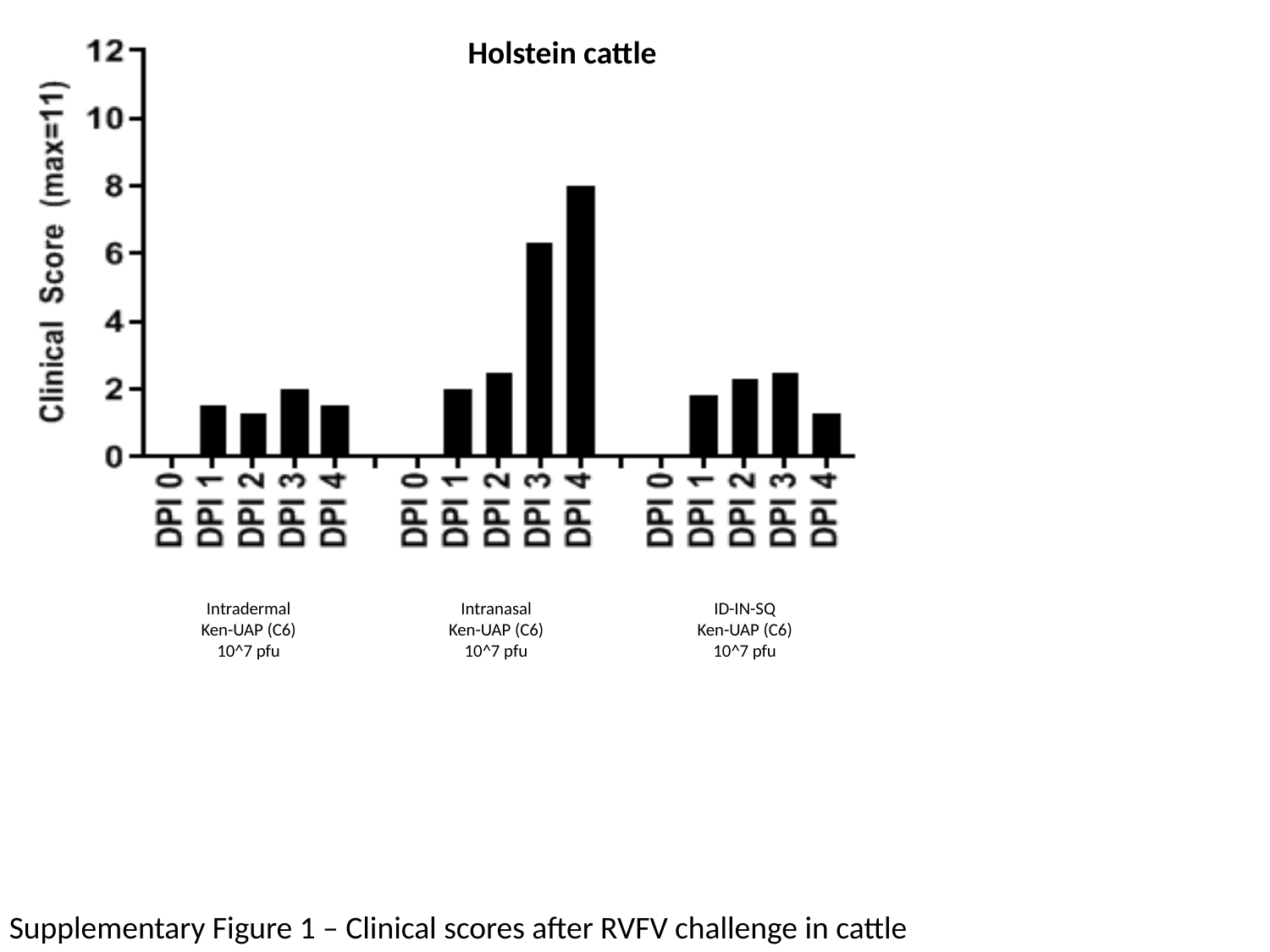

Holstein cattle
Intradermal
Ken-UAP (C6)
10^7 pfu
Intranasal
Ken-UAP (C6)
10^7 pfu
ID-IN-SQ
Ken-UAP (C6)
10^7 pfu
Supplementary Figure 1 – Clinical scores after RVFV challenge in cattle

## Slide 2
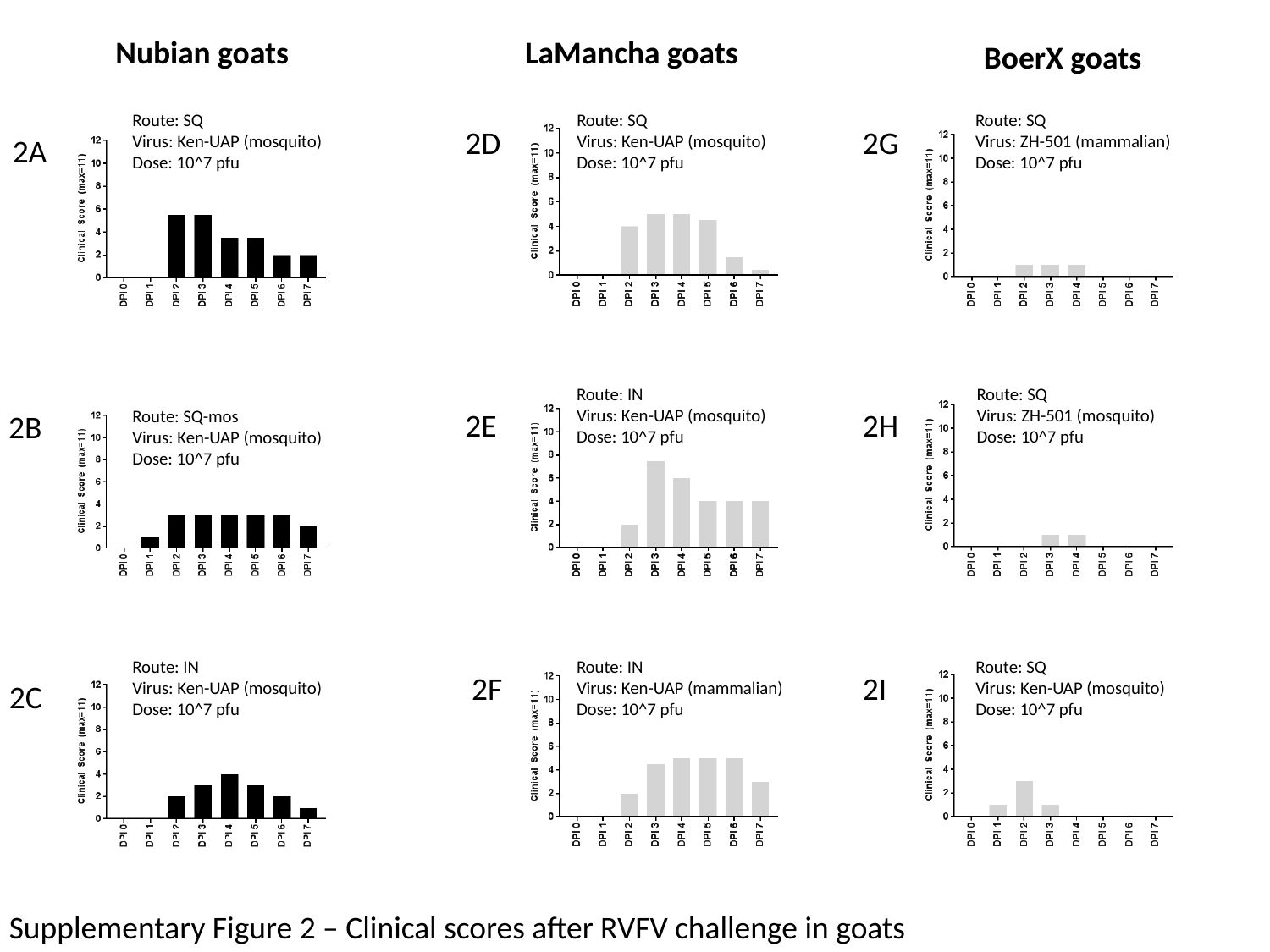

Nubian goats
LaMancha goats
BoerX goats
Route: SQ
Virus: Ken-UAP (mosquito)
Dose: 10^7 pfu
Route: SQ
Virus: Ken-UAP (mosquito)
Dose: 10^7 pfu
Route: SQ
Virus: ZH-501 (mammalian)
Dose: 10^7 pfu
2D
2G
2A
Route: IN
Virus: Ken-UAP (mosquito)
Dose: 10^7 pfu
Route: SQ
Virus: ZH-501 (mosquito)
Dose: 10^7 pfu
Route: SQ-mos
Virus: Ken-UAP (mosquito)
Dose: 10^7 pfu
2E
2H
2B
Route: IN
Virus: Ken-UAP (mosquito)
Dose: 10^7 pfu
Route: IN
Virus: Ken-UAP (mammalian)
Dose: 10^7 pfu
Route: SQ
Virus: Ken-UAP (mosquito)
Dose: 10^7 pfu
2F
2I
2C
IN
Ken-UAP (C6)
10^7 pfu
Supplementary Figure 2 – Clinical scores after RVFV challenge in goats
