# Supplementary material for: Livestock Challenge Models of Rift Valley Fever for Agricultural Vaccine Testing
Source: Front Vet Sci. 2020 May 27;7:238. doi: 10.3389/fvets.2020.00238 (PMC7266933; doi:10.3389/fvets.2020.00238)
Supplement: Supplementary file 2 [file Table_1.docx]

**Supplementary Table 1 – A clinical scoring sheet for RVFV infection in ruminants**

|  | | **Animal #** | **Animal #** | **Animal #** | **Animal #** |
| --- | --- | --- | --- | --- | --- |
|  |  |  |  |  |  |
| **Appearance** | | | | | |
| normal | 0 |  |  |  |  |
| mild disease | 1 |  |  |  |  |
| disease | 2 |  |  |  |  |
| Nasal discharge | 0/x |  |  |  |  |
| Cough | 0/x |  |  |  |  |
| Sneezing | 0/x |  |  |  |  |
| Fever (avg >40C) | 0/1 |  |  |  |  |
| **Disposition** | | | | | |
| BAR (bright, alert and responsive) | 0 |  |  |  |  |
| QAR (quiet, alert and responsive) | 1 |  |  |  |  |
| depressed | 2 |  |  |  |  |
| **Eating** | | | | | |
| normal | 0 |  |  |  |  |
| some eating | 1 |  |  |  |  |
| little/no eating | 2 |  |  |  |  |
| **Drinking** | | | | | |
| normal | 0 |  |  |  |  |
| not drinking | 2 |  |  |  |  |
| **Feces** | | | | | |
| stool normal | 0 |  |  |  |  |
| <50% clumped/soft | 1 |  |  |  |  |
| >50% clumped/soft | 1.5 |  |  |  |  |
| diarrhea (any amount) | 2 |  |  |  |  |
| **MAX SCORE** | **11** |  |  |  |  |
| **Other notes** | | | | | |
|  | | | | | |
